# Rethinking smoking assessment in head and neck squamous cell carcinoma: are we missing something?

**DOI:** 10.3389/fonc.2026.1887251

**Published:** 2026-07-01

**Authors:** Riccardo Gili, Luigi Lorini, Davide Smussi, Armando Di Bello, Paolo Bossi, Carlo Resteghini

**Affiliations:** 1 Medical Oncology and Hematology Unit, IRCCS Humanitas Research Hospital, Milan, Italy; 2 Medical Oncology, Department of Internal Medicine and Medical Specialties, University of Genova, Genoa, Italy; 3 Department of Medical and Surgical Specialties, Radiological Sciences, and Public Health, University of Brescia; Unit of Medical Oncology, Azienda Socio Sanitaria Territoriale (ASST) Spedali Civili, Brescia, Italy; 4 Oncologia Medica, Fondazione Policlinico Universitario Agostino Gemelli Istituto di Ricovero e Cura a Carattere Scientifico (IRCCS), Roma, Italy; 5 Department of Biomedical Sciences, Humanitas University, Milan, Italy

**Keywords:** exposure, HNSCC, packs year smoked, smoking, time

## Abstract

**Introduction:**

Tobacco exposure is a major determinant of risk and clinical outcomes in head and neck squamous cell carcinoma (HNSCC). Although pack-years have long represented the standard metric for quantifying smoking exposure, increasing evidence suggests that this approach incompletely captures the biological and clinical heterogeneity of tobacco-related risk.

**Discussion:**

Recent epidemiological and clinical studies demonstrate that smoking duration rather than its intensity or cumulative pack-years measures is a stronger predictor of HNSCC risk and overall survival, particularly among long-term smokers. Regarding cumulative metrics, metrics like log cigarette-years have shown a better prognostic role when compared with pack-years. Beyond cumulative or duration-based models, smoking cessation and the use of alternative tobacco products substantially influence HNSCC risk and clinical outcomes. While smoking cessation is associated with a progressive reduction in risk and improved survival, active smoking at diagnosis is consistently associated with inferior treatment response. In parallel, non-cigarette tobacco products—including cigars, pipes, and smokeless tobacco—have been shown to independently increase HNSCC risk, are rarely incorporated into exposure models. The growing use of alternative nicotine-containing products, often in combination with cigarette smoking, further complicates risk assessment, as their long-term carcinogenic and immunomodulatory effects remain incompletely characterized.

**Conclusion:**

Pack-years–based models alone are insufficient for accurate risk stratification in HNSCC and realize integrated models including smoking duration assessment may improve prognostic and predictive accuracy, supporting more personalized treatment strategies.

## Introduction

Despite declining smoking prevalence in many high−income countries, tobacco exposure keeps affecting the epidemiology, biology, and outcomes of Head and Neck Squamous Cell Carcinoma (HNSCC) (1). In contemporary cohorts, smoking history influences not only the risk of developing HNSCC but also stage at presentation, comorbidities, treatment tolerance, treatment activity and survival, with particularly strong effects among patients with HPV−negative disease and among HPV−positive patients who continue to smoke (2). For decades, oncologists have relied on pack-years (calculated by multiplying packs smoked per day by years of smoking) as the gold standard for quantifying smoking exposure for HNSCC patients: the historical threshold of ≥10 pack-year has been shown to identify patients at significantly higher risk of adverse outcomes, including reduced overall survival (OS), progression-free survival (PFS), and distant failure–free survival (DFS), and this metric has become deeply embedded in the clinical assessment protocols (3). However, emerging data suggest that alternative thresholds may provide superior prognostic and predictive performance: i.e., Ma et al. have proposed a higher cutoff of 22 pack-year to better discriminate outcomes in selected patient populations (4).

Nevertheless, the use of pack-years does not account for several important factors that proved to impact risk stratification, such as the smoking status – current vs former –, the eventual time since smoking cessation and the use of alternative tobacco or nicotine products (4). Moreover, pack-year metrics homogenizes patients who experienced high-intensity smoking for a limited period with those who smoked for a longer duration at lower intensity, failing to consider smoking intensity and duration as two separate risk factors. Thus, the interest in different multidimensional models which can keep into consideration these aspects. Hereby, we analyze smoking assessment different/complementary to canonical pack-years measurement.

## The evidence for duration-based assessment

Several studies provide evidence for reconsidering the approach to smoking assessment giving more weight to smoking duration. Tomar et al. showed that the duration of tobacco smoking is a stronger risk factor than number of cigarettes smoked per day in HNSCC patients through analysis of pooled case-control data (5). This finding was quantitatively validated by Lam et al. in a large multicenter cohort of 8,875 HNSCC patients, where smoking duration demonstrated an adjusted hazard ratio of 1.11 (95% CI 1.03-1.19) for OS with statistically significant linear dose-response relationships. In addition, the log cig-years measure defined as *log_10_ (cigarettes per day + 1) × years smoked* has shown superior statistical significance when compared to pack-years even when the absolute number of cigarettes smoked is incorporated, even though this metrics is quite more complex to be calculated (6).

Notably, the predominance of exposure duration remained consistent across varied clinical contexts. Duration-based measures surpassed pack-years indices in predicting OS independent of patient age, smoking status, or tumor stage, and they preserved strong performance across anatomic subsites, including the lip and oral cavity, the larynx, and human papillomavirus (HPV)–negative oropharyngeal regions (6).

However, available evidence indicates that the temporal dimension of tobacco exposure does not confer a uniform risk signal across all smoking histories. In light smokers with fewer than 20 years of exposure, prior studies have shown no appreciable increase in HNSCC risk attributable solely to duration. In contrast, risk appears to rise with prolonged exposure, with the highest risk estimates observed among individuals who have smoked for more than 40 years. Taken together, these findings suggest that duration is an unreliable indicator of risk in short-term, low-intensity smokers, yet it becomes a robust and clinically meaningful metric among those with long-standing tobacco use (5–7).

## Biological rationale and clinical implications of duration-based metrics

The performance of duration-based smoking metrics is supported by both biological principles and empirical evidence. Carcinogenesis is inherently a time-dependent process, requiring the sequential accumulation of genetic mutations, sustained epigenetic remodeling, and progressive disruption of cellular homeostasis across many generations of epithelial turnover. Prolonged exposure duration therefore provides the temporal window necessary for cells to go through multiple stages of malignant transformation, making duration a particularly relevant indicator of cumulative carcinogenic injury (6). In addition, persistent tobacco exposure can promote an exhausted T−cell phenotype, which reduces antigen presentation capacity, and alters cytokine profiles potentially modulating sensitivity to immune checkpoint blockade and other immunomodulatory strategies. From a clinical standpoint, adopting duration-based assessment is both practical and impactful. Integration into electronic health records would require only minor adjustments, as duration fields can be added alongside pack-years calculations.

A complementary consideration is the potential value of integrating quantitative cigarette consumption with smoking duration, particularly among individuals who are not heavy smokers. While duration captures the dominant temporal component of carcinogenesis, light or intermittent smokers may accumulate risk in ways that duration alone cannot fully reflect. In these cases, adding a logarithmic intensity measure can refine risk estimation by capturing meaningful variation in low-level exposure without overemphasizing high-intensity consumption. Thus, duration and log-based intensity metrics should be viewed as complementary rather than competing, offering a more complete assessment of tobacco-related risk across diverse smoking patterns.

## Smoking status and the impact on treatment response and clinical outcomes

While the duration of tobacco exposure is a determinant of HNSCC risk, it is equally true that the length of time since smoking cessation carries substantial prognostic relevance when HNSCC is established. Active smoking at the time of diagnosis is consistently associated with poorer clinical outcomes, particularly in terms of treatment responsiveness. Ongoing tobacco use aggravates tumor hypoxia, thereby reducing the effectiveness of radiotherapy and concomitant systemic treatments. This effect is especially pronounced in oropharyngeal cancers, where radiotherapy plays a central role in both definitive and de-intensified treatment strategies. In this disease subset, risk stratification is predominantly based on HPV status. However, beyond the well-established low- and high-risk categories, an intermediate-risk group has been consistently identified, comprising HPV-positive patients with a history of tobacco smoking.

In this context, research efforts have predominantly focused on low-risk patient groups, with comparatively less attention dedicated to high-risk populations. Intermediate-risk cases remain insufficiently characterized and are often not distinctly addressed in current stratification frameworks (8).

Recent advances in functional imaging further strengthen this perspective. The introduction of hypoxia-targeted imaging with ^18F-fluoromisonidazole (FMISO) PET has enabled non-invasive, dynamic assessment of tumor hypoxia, a key determinant of radiotherapy resistance. Importantly, FMISO uptake has been shown to correlate with smoking-related hypoxic patterns, providing a biologically grounded tool for identifying tumors that are intrinsically less sensitive to radiation (9).

The influence of smoking status, however, is not confined to radiotherapy outcomes. Emerging evidence indicates that both current and former smoking can also modulate response to systemic therapies, including immunotherapy. Notably, the KEYNOTE-689 trial, which evaluated neoadjuvant pembrolizumab in resectable HNSCC, demonstrated a greater therapeutic benefit among smokers compared with never-smokers, with the most pronounced effect observed in current smokers (10). Although tobacco exposure is widely associated with systemic and local immunosuppressive effects, the enhanced responsiveness to immune checkpoint inhibition suggests a more complex interaction between smoking-related immune modulation and tumor immunogenicity, which warrants further mechanistic investigation.

Finally, smoking-related cardiopulmonary disease and other comorbidities not only limit the tolerability of intensive multimodality treatments, but also negatively influence anesthetic risk during TORS and other complex procedures, while shaping long-term competing mortality.

Epidemiological evidence further shows that HNSCC risk declines after smoking cessation, indicating that although longer exposure duration reflects greater cumulative carcinogenic damage, substantial risk reduction remains achievable after smoking has stopped (11). Despite these observations, available evidence lacks integration of smoking cessation and its timing into duration-based or cumulative exposure models. Existing studies do not clarify how current abstinence interacts with prior smoking habits, leaving a key gap for clinical decision-making. Determining whether long-duration smokers retain higher residual risk after quitting, or whether cessation provides comparable benefits across duration strata, is crucial for more accurate and personalized counselling (11).

## Future directions and research needs

Although current evidence supports the use and integration of duration-based smoking assessment, several areas of uncertainty require focused investigation. First, it remains unclear whether the impact of long-term exposure varies across demographic subgroups -particularly between men and women, who differ in tobacco metabolism, hormonal influences, and susceptibility to carcinogenic injury. A second priority concerns the integration of smoking cessation into duration-driven models. While quitting reduces risk and improves clinical outcomes, current evidence does not clearly describe how abstinence and its timing interacts with the total years of exposure. Determining whether long-duration smokers retain elevated residual risk even after prolonged cessation - or whether benefits are comparable across duration strata - would directly inform patient counselling and survivorship planning.

Another key gap is to better characterize exposure heterogeneity within long-duration smokers. Variations in smoking intensity, intermittent cessation, and dual use of other tobacco or nicotine products may significantly modulate cumulative carcinogenic effects.

It is important to quantify even non−cigarette tobacco products. Cigars, pipes, and smokeless tobacco remain underrecognized contributors to HNSCC risk. Pooled data from the INHANCE consortium demonstrated that exclusive cigar or exclusive pipe use, in the absence of cigarette smoking, is independently associated with increased HNSCC risk, with odds ratios around 3.5–3.7 compared with never−users (12). Smokeless tobacco, such as chewing tobacco, is strongly linked to oral cavity cancers, particularly in regions with high product use, and contributes to an elevated risk of oral and pharyngeal malignancies (12). Furthermore, long-term impact of recent tobacco products which are rapidly spreading among population such as e-cigarettes and vaping devices is still unknown and so will be for many years.

Cannabis smoking has also been investigated as a potential risk factor, although available evidence remains inconsistent. Early case–control studies reported a possible increased risk among marijuana users, with one analysis suggesting a 2.6-fold higher risk and evidence of dose–response relationships with frequency and duration of use (13). However, subsequent population-based studies and meta-analyses have generally failed to confirm a significant association between lifetime cannabis use and HNSCC development (14, 15). More recently, a large cohort study reported an association between cannabis-related disorders and HNSCC incidence, although causal mechanisms and dose–response relationships remain unclear (16). Overall, current evidence is limited and heterogeneous, and further well-controlled studies are needed to clarify the potential role of cannabis smoking in HNSCC carcinogenesis.

In this context, reliance on pack-years alone may be insufficient to capture the biological and clinical heterogeneity of HNSCC patients and specifically of HPV-positive patients with smoking history. Incorporating additional dimensions of tobacco exposure - specifically smoking duration, current smoking status, time from cessation and the concomitant or alternative use of other tobacco/nicotine products - could enable a more refined stratification of risk and treatment sensitivity. Accounting for not only cumulative exposure but also the temporal dynamics of smoking behavior may allow the identification of distinct subgroups with differential radiosensitivity and clinical outcomes, thereby supporting more individualized treatment approaches (8).

Taken together, these observations underscore that smoking-related risk cannot be adequately characterized by quantity alone, and the integration of other parameters may be particularly relevant in terms of prognosis and selection among treatment modalities, such as radiotherapy intensification, treatment de-escalation in HPV-positive disease, or the incorporation of immunotherapy.

Finally, mechanistic studies are needed to elucidate the relationship between duration and outcomes. Investigations linking exposure duration with mutational burden, epigenetic remodeling, tumour hypoxia, and other biological markers could reveal the pathways through which long-term smoking shapes both carcinogenesis and treatment response. Such insights may ultimately guide personalized therapeutic strategies.

Overall, advancing these research fronts will be critical to developing more accurate, biologically grounded, and clinically actionable models of smoking exposure in HNSCC.

## Conclusion

Cumulative smoking models alone are insufficient to accurately stratify risk and prognosis in HNSCC. Alternative metrics, such as log cigarette-years, may provide improved prognostic discrimination compared with traditional pack-years-based measures, but are to date poorly used since they are more complex. Indeed, smoking duration is a simple data that captures relevant aspects of exposure not fully reflected by cumulative quantity alone and therefore should be incorporated into integrated prognostic and predictive models. Further large, multicenter studies involving well-characterized patient cohorts are needed to develop more refined prognostic and predictive models than those currently available and therefore to support more personalized treatment strategies.

## Data Availability

The original contributions presented in the study are included in the article/supplementary material. Further inquiries can be directed to the corresponding author.
